# Supplementary material for: Protein structural biology using cell-free platform from wheat germ
Source: Adv Struct Chem Imaging. 2018 Nov 10;4(1):13. doi: 10.1186/s40679-018-0062-9 (PMC6244559; doi:10.1186/s40679-018-0062-9)
Supplement: Supplementary file 1 — Additional file 1. Additional discussion, figures, table. [file 40679_2018_62_MOESM1_ESM.docx]

**ADDITIONAL INFORMATION FOR**

**Protein structural biology using cell-free platform from wheat germ**

Irina V. Novikova^1^**,** Noopur Sharma^1^, Trevor Moser^1^, Ryan Sontag^1^, Yan Liu^2^, Michael J. Collazo^3,4^, Duilio Cascio^3.4^, Tolou Shokuhfar^5^, Hanjo Hellmann^2^, Michael Knoblauch^2^ & James E. Evans^1,2*^

**Affiliations:**

1. Environmental Molecular Sciences Laboratory, Pacific Northwest National Laboratory, 3335 Innovation Blvd., Richland, WA, 99354, USA
2. School of Biological Sciences, Washington State University, Pullman, WA, 99164, USA
3. Department of Biological Chemistry, University of California Los Angeles, Howard Hughes Medical Institute, UCLA-DOE Institute for Genomics and Proteomics, Los Angeles, CA, 90095, USA
4. Department of Chemistry and Biochemistry, University of California Los Angeles, Howard Hughes Medical Institute, UCLA-DOE Institute for Genomics and Proteomics, Los Angeles, CA, 90095, USA
5. Department of Bioengineering, University of Illinois at Chicago, Chicago, IL, 60607, USA

**Emails:** [irina.novikova@pnnl.gov](mailto:irina.novikova@pnnl.gov), [noopur.sharma@pnnl.gov](mailto:noopur.sharma@pnnl.gov), [trevor.moser@pnnl.gov](mailto:trevor.moser@pnnl.gov), [ryan.sontag@pnnl.gov](mailto:ryan.sontag@pnnl.gov), [yan.liu4@wsu.edu](mailto:yan.liu4@wsu.edu), [tolou@uic.edu](mailto:tolou@uic.edu), [knoblauch@wsu.edu](mailto:knoblauch@wsu.edu), [hellmann@wsu.edu](mailto:hellmann@wsu.edu), [cascio@mbi.ucla.edu](mailto:cascio@mbi.ucla.edu), [mcollazo@mbi.ucla.edu](mailto:mcollazo@mbi.ucla.edu)

**The email of corresponding Author(s):** [james.evans@pnnl.gov](mailto:james.evans@pnnl.gov)

**Included:**

Additional Discussion

Additional Figures 1-4

Additional Table 1

**Discussion**

**Nested PCR to fish out the "hard-to-amplify" genes from genomic DNA.** While the GBBSI protein was easily amplified specifically from the genome of *O. tauri*, direct amplification of genes of interest from genomic DNA can often fail for many reasons. The complexity and the size of the genome, repetitive regions, non-optimal primer design are among some of the defining factors [1]. We encountered such failures for the glutamine synthetase (GS, 75 kDa) and pyruvate, phosphate dikinase (PPDK, 100 kDa) genes that we tried to amplify directly from the genome of *O. tauri.* To increase our chances for success, we employed a nested PCR strategy [2]. The idea of nested PCR (Figure S4) is that one set of primers is used to first amplify a larger portion of the genome containing your gene of interest. Depending on the genome complexity and size, it is common that the desired band will not even be visible due to nonspecific priming events. Nevertheless, the resulting PCR library exhibits much less complexity and is further subjected to a second round of PCR using a pair of the gene-specific primers to amplify the target gene alone. The latter approach significantly increases the specificity of DNA amplification, and thus it has been used extensively for viral detection in clinical samples and in mutation screening assays [2-4].

  Use of only one pair of nested FWD_N1 and REV_N1 primers proved sufficient to amplify GS (Figure S4B). A wide range of PCR products is observed in the first round, while at the second round only one higher molecular weight DNA band of the desired length emerged. The DNA band was further excised, gel-purified and used in the Gibson Assembly with the linearized pEU plasmid to test for vector-based cell-free translation. The specific amplification was confirmed by the sequencing of clones. We also tested this approach to amplify another "difficult" gene - PPDK. The GC content reaches 73% in the upstream region and N-terminal portion of the PPDK gene, which gives poor prognosis for its amplification. Indeed, multiple efforts to amplify PPDK directly failed. Therefore, in the nested primer design, we also included an additional nested FWD_N3 primer in order to generate two additional PCR libraries (Figure S4C). Quite diverse PCR populations were generated in the first round of PPDK amplification. They were further subjected to a second round of PCR using gene-specific primers, and four out of six libraries generated similar higher molecular weight products of expected to 4.4 kB size. Subsequent purification and sequencing of these products confirmed that PPDK gene was successfully amplified from N2/N2 library.

1. Andreson, R., Mols, T., Remm, M.: Predicting failure rate of PCR in large genomes. Nucleic acids research 36(11), e66 (2008). doi:10.1093/nar/gkn290
2. Yourno, J., Conroy, J.: A novel polymerase chain reaction method for detection of human immunodeficiency virus in dried blood spots on filter paper. J Clin Microbiol 30(11), 2887-2892 (1992).
3. Rabodonirina, M., Raffenot, D., Cotte, L., Boibieux, A., Mayencon, M., Bayle, G., Persat, F., Rabatel, F., Trepo, C., Peyramond, D., Piens, M.A.: Rapid detection of Pneumocystis carinii in bronchoalveolar lavage specimens from human immunodeficiency virus-infected patients: Use of a simple DNA extraction procedure and nested PCR. Journal of Clinical Microbiology 35(11), 2748-2751 (1997).
4. Michalska, D., Jaguszewska, K., Liss, J., Kitowska, K., Mirecka, A., Lukaszuk, K.: Comparison of whole genome amplification and nested-PCR methods for preimplantation genetic diagnosis for BRCA1 gene mutation on unfertilized oocytes-a pilot study. Hered Cancer Clin Pr 11 (2013). doi:10.1186/1897-4287-11-10

**Additional Figures**


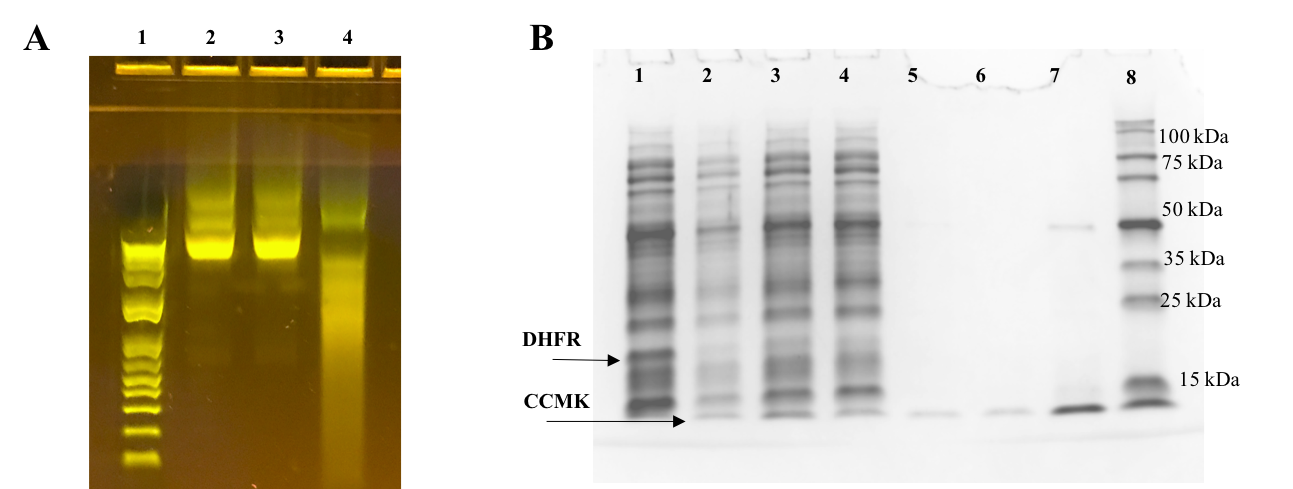


Figure S1. A. The detection of various mRNAs on agarose. Lane 1, MW DNA markers; Lanes 2-3, good quality mRNAs; Lane 4: low quality mRNA. B. SDS-PAGE gel, showing the expression and purification of CCMK protein using 6xHis-tag and wash buffer supplemented with 10 mM imidazole. Lane 1, control DHFR expression; Lane 2, crude mixture; Lane 3, soluble fraction; Lane 4, flow-through; Lane 5, elution fraction 1; Lane 6, elution fraction 2; Lane 7, elution fractions 1 and 2 combined and concentrated; Lane 8, MW markers.


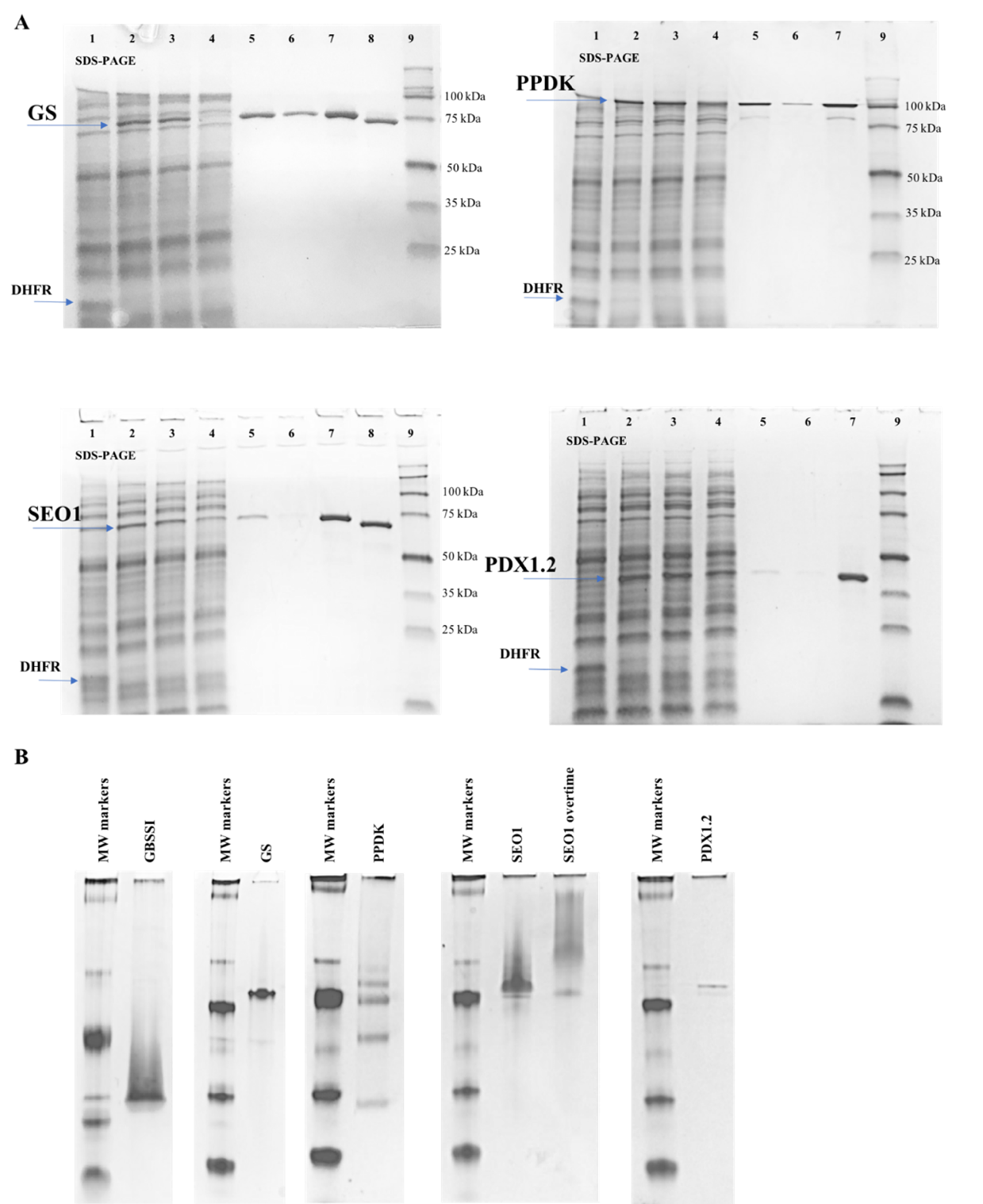


Figure S2. Protein purification and analysis by PAGE. A. SDS-PAGE gel of expression and purification of various proteins using 3XFLAG-tag. Lane 1, control DHFR expression; Lane 2, crude mixture; Lane 3, soluble fraction; Lane 4, flow-through; Lane 5, elution fraction 1; Lane 6, elution fraction 2; Lane 7, elution fractions 1 and 2 combined and concentrated; Lane 8, 3XFLAG tag removed; Lane 9, MW markers. B. Native PAGE analysis with NativeMark molecular weight standards shown for each panel. For SEO1, lanes are shown of the freshly expressed protein (SEO1) as well as following 3XFLAG removal and additional incubation time to show the shift towards higher molecular weight assemblies (SEO1 overtime).

**pEU_3XFLAG_ gene:**

5’…..ATTTAGGTGACACTATAGAACTCACCTATCTCCCCAACACCTAATAACATTCAATCACTCTTTCCACTAACCACCTATCTACATCACCAATGGACTACAAGGACCATGATGGCGATTATAAGGATCACGATATAGACTACAAAGATGATGACGACAAGCTCGCTgeneTAAGGATCCATATATAGGGCCCGGGTTATAATTACCTCAGGTCGACGTCCCATGGTTTTGTATAGAATTTACGGCTAGCGCCGGATGCGACGCCGGTCGCGTCTTATCCGGCCTTCCTATATCAGGCGGTGTTTAAGACGCCGCCGCTTCGCCCAAATCCTTATGCCGGTTCGACGACTGGACAAAATACTG….3’

**pEU_gene_6xHis:**

5’…..ATTTAGGTGACACTATAGAACTCACCTATCTCCCCAACACCTAATAACATTCAATCACTCTTTCCACTAACCACCTATCTACATCACCAATGGACTACAAGGACCATGgeneCATCATCATCATCATCATTAAGGATCCATATATAGGGCCCGGGTTATAATTACCTCAGGTCGACGTCCCATGGTTTTGTATAGAATTTACGGCTAGCGCCGGATGCGACGCCGGTCGCGTCTTATCCGGCCTTCCTATATCAGGCGGTGTTTAAGACGCCGCCGCTTCGCCCAAATCCTTATGCCGGTTCGACGACTGGACAAAATACTG….3’

**pEU_3XFLAG_ gene_6xHis:**

5’…..ATTTAGGTGACACTATAGAACTCACCTATCTCCCCAACACCTAATAACATTCAATCACTCTTTCCACTAACCACCTATCTACATCACCAATGGACTACAAGGACCATGATGGCGATTATAAGGATCACGATATAGACTACAAAGATGATGACGACAAGCTCGCTgeneCATCATCATCATCATCATTAAGGATCCATATATAGGGCCCGGGTTATAATTACCTCAGGTCGACGTCCCATGGTTTTGTATAGAATTTACGGCTAGCGCCGGATGCGACGCCGGTCGCGTCTTATCCGGCCTTCCTATATCAGGCGGTGTTTAAGACGCCGCCGCTTCGCCCAAATCCTTATGCCGGTTCGACGACTGGACAAAATACTG….3’

Figure S3. The sequence design of pEU constructs used in this study.

**
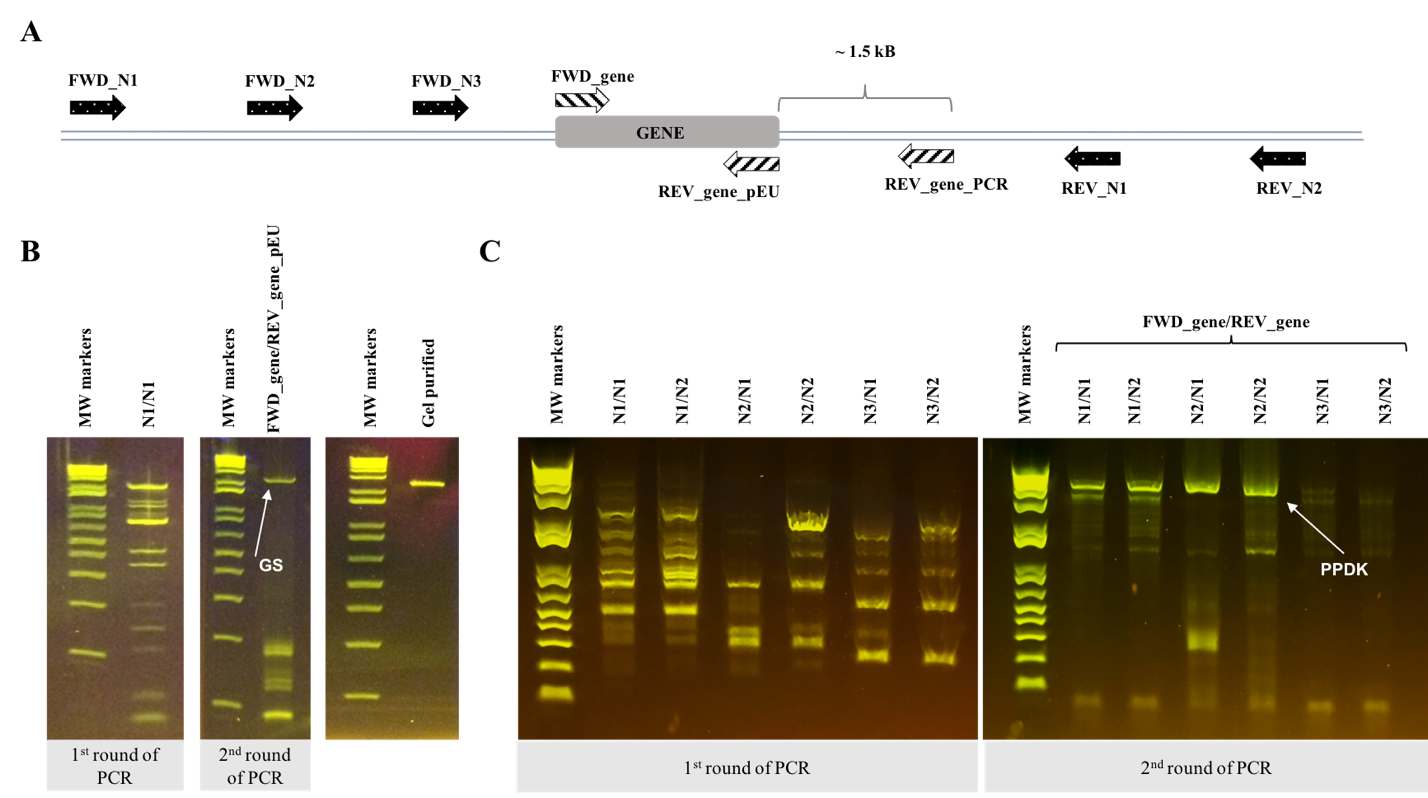
**

Figure S4. Nested PCR approach for “hard-to-amplify” genes from the genomic DNA. A. A general primer design for nested PCR. First round of PCR uses primers denoted with _N and filled with black. They should bind 0.5kB-1.5kB upstream and downstream from the desired gene. The second round of PCR employs two on-gene primers: FWD_gene and REV_gene_for_pEU primers for insertion in pEU plasmid or FWD_gene and REV_gene_PCR for PCR template. B. Nested PCR amplification of Glutamine Synthetase (GS) gene from the genome of *O. tauri*. During the first round of PCR (the agarose gel on the left), a diverse library of PCR products was generated. The second round of PCR on obtained N1/N1 library using gene-specific primers resulted in the specific amplification of GS gene (the agarose gel in the middle). C. Nested PCR amplification of Pyruvate, Phosphate Dikinase (PPDK) gene from the genome of *O. tauri*. The agarose gel on the left shows the PCR products obtained using first round of PCR. These PCR products were subjected to the second round of PCR using gene-specific primers, which yielded the specific amplification (the agarose gel on the right).

**Additional Tables**

| **Data Collection** |  |
| --- | --- |
| **Beamline** | APS-NECAT 24ID-C |
| **Detector** | Pilatus 6MF pixel array |
| Space group | P1 |
| Unit cell dimensions |  |
| *a,b,c*, (Å) | 144.01,144.43,147.0 |
| α,β,γ (°) | 116.87, 112.19,85.28 |
| Reflections observed | 60003 |
| Unique reflections | 30307 |
| Wavelength (Å) | 0.9792 |
| Resolution (Å) | 78.5 -5.5 |
| Highest Resolution Shell (Å) | 5.6 -5.5 |
| R_sym_ (%)^a,b^ | 11.9(71.9) |
| CC(1/2) | 98.6(55.1) |
| I/σ | 3.89(1.00) |
| Completeness (%) | 95.3(94.4) |
| Wilson B value (Å^2^) | 224.9 |

^a^ Highest resolution shell shown in parenthesis

^b^. R_sym_ = Σ | I-<I> | / Σ I

Table S1. X-ray macromolecular crystallography data collection and preliminary refinement statistics for GS complex, generated via cell-free pipeline.
